# Eupafolin Suppresses Esophagus Cancer Growth by Targeting T-LAK Cell-Originated Protein Kinase

**DOI:** 10.3389/fphar.2019.01248

**Published:** 2019-10-22

**Authors:** Xiaoming Fan, Junyan Tao, Xin Cai, Mangaladoss Fredimoses, Junzi Wu, Zhihui Jiang, Kunpeng Zhang, Shude Li

**Affiliations:** ^1^Henan Joint International Research Laboratory of Veterinary Biologics Research and Application, Anyang Institute of Technology, Anyang, China; ^2^Institute of Environmental Safety and Human Health, Wenzhou Medical University, Wenzhou, China; ^3^Laboratory of Natural Product Extraction, China-US (Henan) Hormel Cancer Institute, Zhengzhou, China; ^4^College of Basic Medical, Yunnan University of Chinese Medicine, Kunming, China; ^5^Department of Biochemistry and Molecular Biology, School of Basic Medicine, Kunming Medical University, Kunming, China; ^6^Yunnan Province Key Laboratory for Nutrition and Food Safety in Universities, Kunming, Yunnan, China

**Keywords:** eupafolin, esophagus cancer, TOPK, inhibitor, Ay Tsao

## Abstract

Eupafolin is the main bioactive component extracted from the traditional Chinese medicine Ay Tsao (*Artemisia vulgaris* L.), and its anti-tumor activity has had been studied in previous researches. T-LAK cell-originated protein kinase (TOPK) belongs to serine/threonine protein kinase and is highly expressed in several cancer cells and tissues, such as colon cancer, lung cancer, esophagus cancer, and so on. Therefore, it was recognized as an important target for treating tumors. Nowadays, we found that eupafolin suppressed TOPK activities at the first time *in vitro* and *in vivo*. The cells study indicated that eupafolin suppressed TOPK activities in JB6 Cl41 and KYSE450 cells. Furthermore, knockdown of TOPK in KYSE450 cells decreased their sensitivities to eupafolin. The animal study showed that the injection of eupafolin in patient-derived xenograft (PDX) mouse effectively suppressed tumor growth. Histone H3 and Ki67 were reduced, and cleaved caspase 3 was increased in tumor tissues after eupafolin treatment. To sum up, eupafolin as an TOPK inhibitor can suppress growth of esophagus cancer *in vitro* and *in vivo*. The TOPK downstream signaling molecule histone H3 in tumor tissues was also reduced after eupafolin treatment. In short, eupafolin can suppress growth of esophagus cancer cells as an TOPK inhibitor both *in vitro* and *in vivo*.

## Introduction

T-LAK cell-originated protein kinase (TOPK) is a serine-threonine kinase, which is a member of MAPKK family. TOPK is also related with the mitotic spindle to the centromere. The studies showed that over-expression of TOPK leads to characteristic of cancerous cell, creating aneuploidy cells in transformed cells JB6 C141 cells (Zhu et al., 2007). TOPK was highly expressed in several malignancies and promoted tumorigenesis and progression (Herbert et al., 2018; Xu and Xu, 2019). Therefore, TOPK might be an excellent drug target for cancer chemotherapy.

It is necessary to find TOPK inhibitors with low toxicity to overcome the side-effect of current TOPK inhibitors (Matsuo et al., 2014; Ishikawa et al., 2018). There were several small molecule compounds from Chinese herbal medicine reported to be an inhibitor of TOPK to reduce the proliferation of tumor cells (Diao et al., 2019; Wang et al., 2019). However, the study of inhibiting TOPK in esophageal cancer has not been reported, although TOPK was highly expressed in esophageal cancer.

In our study, we found that eupafolin can block TOPK and inhibit TOPK kinase activity.

## Materials and Methods

### Reagents and Antibodies

Eupafolin or 6-methoxyluteolin was extracted from Ay Tsao (*Artemisia vulgaris* L.). The plant material was purchased from Anyang Jiutou Xian Ai Co. Ltd. (Anyang, China). Air-dried plant material (3.0 kg) of Ay Tsao was extracted with 95% ethanol under reflux three times, for 2 h for each time. The extract was concentrated in rotary vacuum evaporator to give a residue (23.5 g). The residue was dissolved in H_2_O and then extracted successfully with petroleum ether (2 L), EtOAC (2 L), and n-BuOH (each 2 L). The active EtOAC fraction (15.7 g) was subjected in to silica column chromatography to (200–300 mesh) and eluted with petroleum ether/EtOAc (90:10, 80:20, 50:50, 25:75) and followed by CHCl3/MeOH in a stepwise gradient (90:10, 80:20, 70:30, 60:40, 0:100) to obtain eight fractions (fr.1−8) on the basis of TLC profiles. Fraction 3 (1.785 g) was further chromatographed on Sephadex LH-20 eluted with (CHCl3/MeOH 1:1) to furnish four sub-fractions, designated as fr.3–1 to 3–4. Fr.3–2 (265.2 mg) was further purified by preparatory TLC to obtained pure compound 6-methoxyluteolin (14.8 mg).

HI-TOPK-032 was purchased from National Institutes of Health (NIH). Recombinant active TOPK and inactive TOPK were purchased from Millipore (Billerica, MA). The CNBr-Sepharose 4B was purchased from GE Healthcare (Pittsburgh, PA). Antibodies to detect β-actin, p-histone H3, histone H3, and cleaved caspase-3 were from Cell Signaling Technology (Danvers, MA).

### Cell Culture and Cytotoxicity Assay

The cells were purchased from American Type Culture Collection. They were cultured at 37°C in a 5% CO2 incubator using DMEM medium containing 10% fetal calf serum. Cells were planted in 96 well plant and treated with different doses of eupafolin. The cytotoxicity of eupafolin was measured using 3-(4,5-Dimethylthiazol2-yl)-5-(3-carboxymethoxyphenyl)-2H-tetrazdium (MTS) Assay Kit (Promega, Madison, WI) according to the manufacturer’s instructions.

### Soft Agar Assay

The cell lines (8 × 10^3^/well) were suspended in a six-well plate were exposed or not exposed to EGF (20 ng/ml) and cultured in 1 ml of 0.3% Basal Medium Eagle Agar Medium (Sigma–Aldrich Corp.) containing 10% FBS over 3 ml of 0.5% BME agar containing 10% FBS. The cells were maintained about 5–10 days in a 37°C and 5% CO2 incubator, and then, their colonies were counted by microscopy.

### Molecular Docking Model

To estimate the interaction mode of TOPK and eupafolin, a TOPK structure was modeled and subsequent induced-fit docking was applied. Three-dimensional protein model of TOPK (5j0a) was downloaded from the Protein Data Bank (PDB). Among those with the highest sequence identity (30%) with TOPK, structures of 4L52, 2EVA, and 4GS6 (PDB entries) were protein-ligand complex, thus suitable for the modeling of the TOPK and eupafolin complex. The sequence of TOPK and the four templates, 4L52, 2EVA, 4GS6 and 2F4J, were aligned using SYBYL-X 2.0 server with the default parameters.

### Microscale Thermophoresis (MST)

Inactive TOPK protein was labeled with the Monolith NT^™^ Protein Labeling Kit RED (Cat#L001) according to the supplied labeling protocol. The TOPK proteins were diluted in a 20 mM HEPES (pH 7.4) and 0.05 (v/v) % Tween-20 to 50 nM. The eupafolin stock was dissolved in ddH_2_O in a concentration of 5 mM. We used 5 mM eupafolin as the highest concentration for the serial dilution. After 10 min incubation at room temperature, the samples were loaded into Monolith^™^ standard-treated capillaries, and the thermophoresis was measured at 25°C after 30 min incubation on a Monolith NT.115 instrument (NanoTemper Technologies, München, Germany). Laser power was set to 20% or 40% using 30 s on time. The LED power was set to 100%. The dissociation constant Kd values were fitted by using the NTAnalysis Software (NanoTemper Technologies, München, Germany) (Wienken et al., 2010).

### *In Vitro* Beads Binding Assay

KYSE450 cell lysates (1 mg) were incubated with eupafolin-Sepharose 4B beads in the reaction buffer [5 mM ethylenediaminetet acid, 150 mM NaCl, 50 mM Tris (pH 7.5), 1 mM dithiothreitol, 2 µg/ml bovine serum albumin, 0.01% Nonidet P-40, 1 µg/ml protease inhibitor mixture, and 0.02 mM phenylmethylsulfonyl fluoride]. After incubation with gentle rocking overnight at 4°C, the beads were washed five times, and proteins bound to the beads were detected by western blotting.

### Protein Expression and Purification of the GST-Histone H3

The human GST-histone H3 fusion protein was expressed in *Escherichia coli* BL21 bacteria. The bacteria were grown at 37°C to an absorbance of 0.8–0.9 at 600 nm, induced with 0.5 mM isopropyl-β-D-thiogalactopyranoside (IPTG) 2–3 h at 37°C, and then harvested by centrifugation. The cell pellets were suspended in phosphate buffered saline (PBS). After sonication and centrifugation, the supernatant fraction was incubated with Glutathione-Sepharose beads (GE, USA) overnight at 4°C. The beads were washed with PBS and then eluted with 50 mM glutathione. After protein quantitation, the samples were separated by a 10% SDS-PAGE and visualized by Coomassie brilliant blue staining.

### *In Vitro* Kinase Assay

The TOPK active kinase (0.2 μg) and inactive GST- histone H3 substrate (2 μg) were incubated at 32°C for 40 min in 1×kinase buffer (25 mM Tris-HCl pH 7.5, 5 mM beta- glycerophosphate, 2 mM dithiothreitol, 0.1 mM Na3VO4, 10 mM MgCl2, and 5 mM MnCl_2_) containing 100 μM ATP. The samples were added with 5×SDS buffer and detected by western blot.

### Western Blot

The cells were harvested with 300 μl of RIPA buffer and sonicated 15 s for three times and centrifuged at 13,000 rpm for 10 min. Then the quantity of protein was determined by the BCA method. The samples (30 μg) with 5×SDS loading buffer were heated at 95°C for 10 min and then separated on a 10%-15% SDS-PAGE and subsequently transferred onto a PVDF membrane, then the membrane was blocked with 5% milk for 1 h and added into special primary antibody at 4°C overnight. Then, the membrane was washed 5 min for three times, and added secondary antibody was labeled with HRP. The membrane was detected by chemiluminescence.

### Patient-Derived Xenograft (PDX) Mouse Model

Esophageal cancer (Anyang Tumor Hospital) fragments (2–3 mm) were implanted into the immune deficient (SCID) mice. Mice were divided into three groups: control group and two eupafolin-treated (20 or 50 mg/kg) groups. Five days after tumor implantation, mice were treated with control (0.9% saline) or eupafolin by i.p. injection three times a week for 35 days. Body weight and tumor volume were measured once a week, tumor volume was calculated using the formula, tumor volume = length×width×height×0.5. Tumor tissues and peritumoral tissues were embedded in a paraffin block and stained by immunohistochemistry. This study was approved under a protocol approved by the Anyang Institute of Technology (Anyang, Henan, China).

### Immunohistochemistry Staining

Then, the sections were incubated at 4°C overnight with an antibody against histone H3 and cleaved caspase-3 (diluted 1:200). Then, sections were washed in phosphate-buffered saline (PBS) and incubated with the secondary antibody (biotinylated goat anti-rabbit, 1:200; Vector Laboratories, Burlingame, CA) for 30 min. The sections were counterstained with hematoxylin after diaminobenzidine staining. Photomicrographs were taken with a digital camera. The positively stained cells within each photomicrograph were counted.

### Caspase-3 Activity Assay

Caspase-3 activity in the tumor and peritumoral tissues was determined by a Caspase-3 Activity Assay Kit (BioVision K106; BioVision, Milpitas, CA, USA) according to the manufacturer’s instructions. Tissues were ground and then incubated with cold lysis buffer on ice for 15 min. The lysed tissues were centrifuged for 10 min at 16,000 g at 4°C; then, the supernatants were collected, and the protein concentrations were calculated. The supernatants were transferred to a 96-well plate containing detection buffer, and Ac-DEVD-pNA was added. After incubation at 37°C for 2 h, absorbance was measured at 405 nm with a microplate reader (Thermo Fisher Scientific). The caspase-3 activity of each sample was calculated according to the standard curve and normalized to the protein concentration.

### Statistical Analysis

All quantitative data are expressed as mean values ± standard deviation, and significant differences were determined by Student’s t test or by one-way ANOVA. P < 0.05 was used as the criterion for statistical significance.

### Ethics Statement

Primary tumor samples of ESCC were obtained from 10 consecutive patients with ESCC who had undergone curative esophagostomy at the Division of Digestive Surgery, Department of Surgery, Anyang Tumor Hospital (Anyang, China), between 2017 and 2019. Written consent was always obtained in the formal style and after approval by the local Ethics Committee. None of these patients had undergone endoscopic mucosal resection, palliative resection, preoperative chemotherapy, or radiotherapy, and none of them had synchronous or metachronous multiple cancer in other organs. The animal study was reviewed and approved by Henan Joint International Research Laboratory of Veterinary Biologics Research and Application, Anyang Institute of Technology.

## Results

### Eupafolin Binds With TOPK and Inhibits TOPK Activity

**Figure 1:**
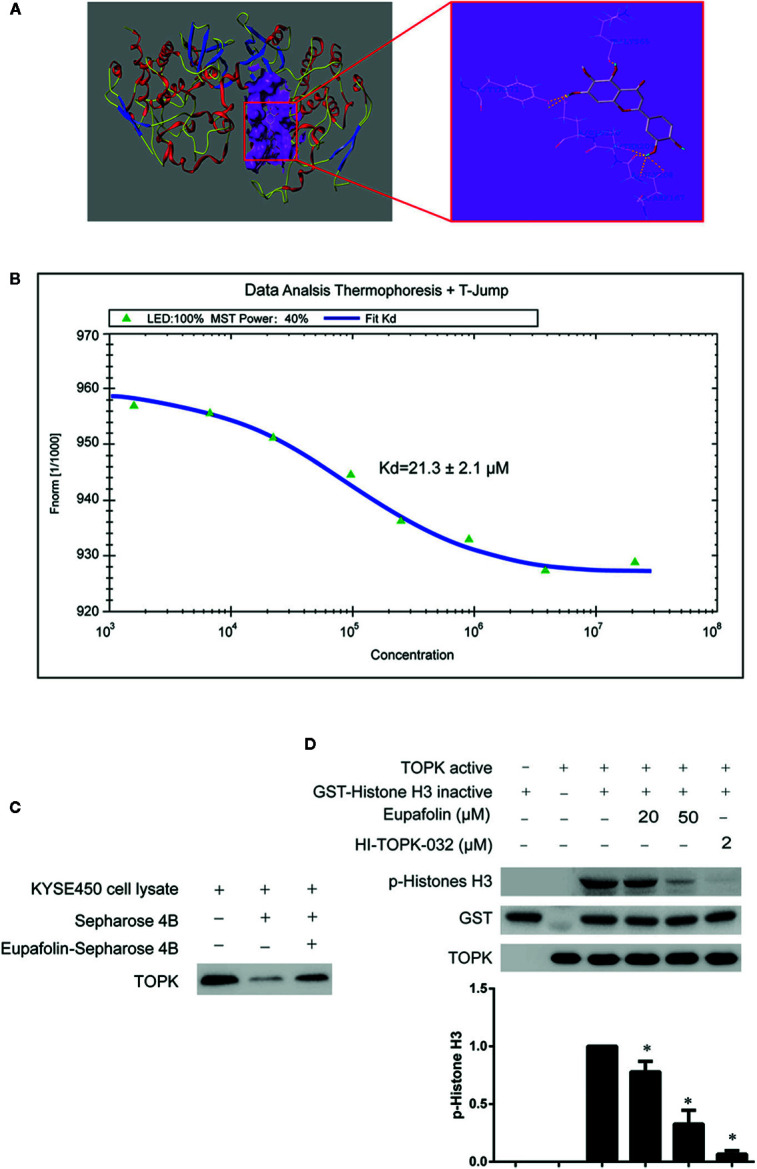
Eupafolin binds with TOPK and suppresses TOPK activity *in vitro*. **(A)** The docking model of eupafolin and TOPK. **(B)** Measurement of affinity between TOPK and eupafolin by MST in standard treated capillaries, and the resulting binding curve was shown. From the resulting binding curve, Kd of 21.3 ± 2.1 is calculated. **(C)** Eupafolin binds directly with TOPK. Sepharose 4B was used for binding and pull-down assay as described in section “Materials and methods.” Lane 1 is input control (TOPK protein standard); lane 2 is the negative control, indicating there is no binding between TOPK and beads alone; and, lane 3 indicates that TOPK binds with eupafolin-Sepharose 4B beads. **(D)** Eupafolin inhibits TOPK activity *in vitro*. The inhibitory effect of eupafolin on TOPK was determined by an *in vitro* kinase assay. An inactive GST-histone H3 protein was used as the substrate with active TOPK and 100 μM ATP in the reaction buffer. Protein were resolved by 10% SDS-PAGE gel and detected by Western blot. Histogram statistics is the expression of the p-histone H3 in the first line. Data are representatives of results from triplicate experiments. *Significant compared with lane 3 alone, P < 0.05.

To estimate whether eupafolin binds to TOPK, the homology modeling and subsequent molecular docking method were applied. The binding model generated by docking simulation indicated that the compound eupafolin was positioned at the hydrophobic pocket of TOPK, surrounded by the residues Tyr-271, Lys-65, Glu-210, Thr-209, and Gly-208, forming a stable hydrophobic binding (**Figure 1A**). To further evaluate this binding model, the MST method can quantify protein and small molecule interactions with high sensitivity and low sample cost by detecting fluorescent changes of molecules during thermophoresis. We detected the binding affinity between several nature compounds and TOPK using this technology. The results showed that the eupafolin had the lowest equilibrium dissociation constant (Kd) of 21.3 ± 2.1 µM (**Figure 1B**, **Table 1**), which meant the strongest binding between the eupafolin and TOPK.

**Table 1:** Binding affinity and inhibitory activities of screening hits.

Compound	ICM docking mf score^a^	Dissociation constant with TOPK	Inhibitory activities against KYSE 450 cells
	(kcal/mol)	Kd^b^ (μM)	IC50 (μM)
Jaceosidin	−87.11	674 ± 12.2	n.i^c^
Bergenin	−146	438 ± 9.5	n.i^c^
Eupafolin	−125	21.3 ± 2.1	145.2
Apigenin	−89.27	384 ± 47.6	n.i^c^

a Docking score/interaction potential of compounds with TOPK (kcal/mol).

b The Kd value is automatic calculated by the curve fitting, and presents as means ± SD.

c n.i. is no inhibition detected in the experiments.

To validate the veracity of the MST method, we employed the *in vitro* beads binding assay to analyze the binding between eupafolin and TOPK in esophageal adenocarcinoma KYSE450 cell lysates which has high expression of TOPK. No obvious band representing TOPK was observed in the beads without eupafolin group, whereas a strong band was seen in eupafolin-conjugated beads group (**Figure 1C**).

The above results implied that eupafolin might inhibit the TOPK activity. To confirm this hypothesis, we performed an *in vitro* kinase assay with inactive GST-histone H3 as the substrate with active TOPK in the presence of 25, 50, and 100 µM of eupafolin and 10 nM ATP. The results showed that phosphorylation of histone H3 (Ser10) was substantially attenuated in a dose-dependent manner after treatment with eupafolin (**Figure 1D**). HI-TOPK-032, a TOPK inhibitor, was used as a positive control (Kim et al., 2012).

### Eupafolin Inhibits EGF-Induced Neoplastic Transformation and Signal Transduction in JB6 Cl41Cells

**Figure 2:**
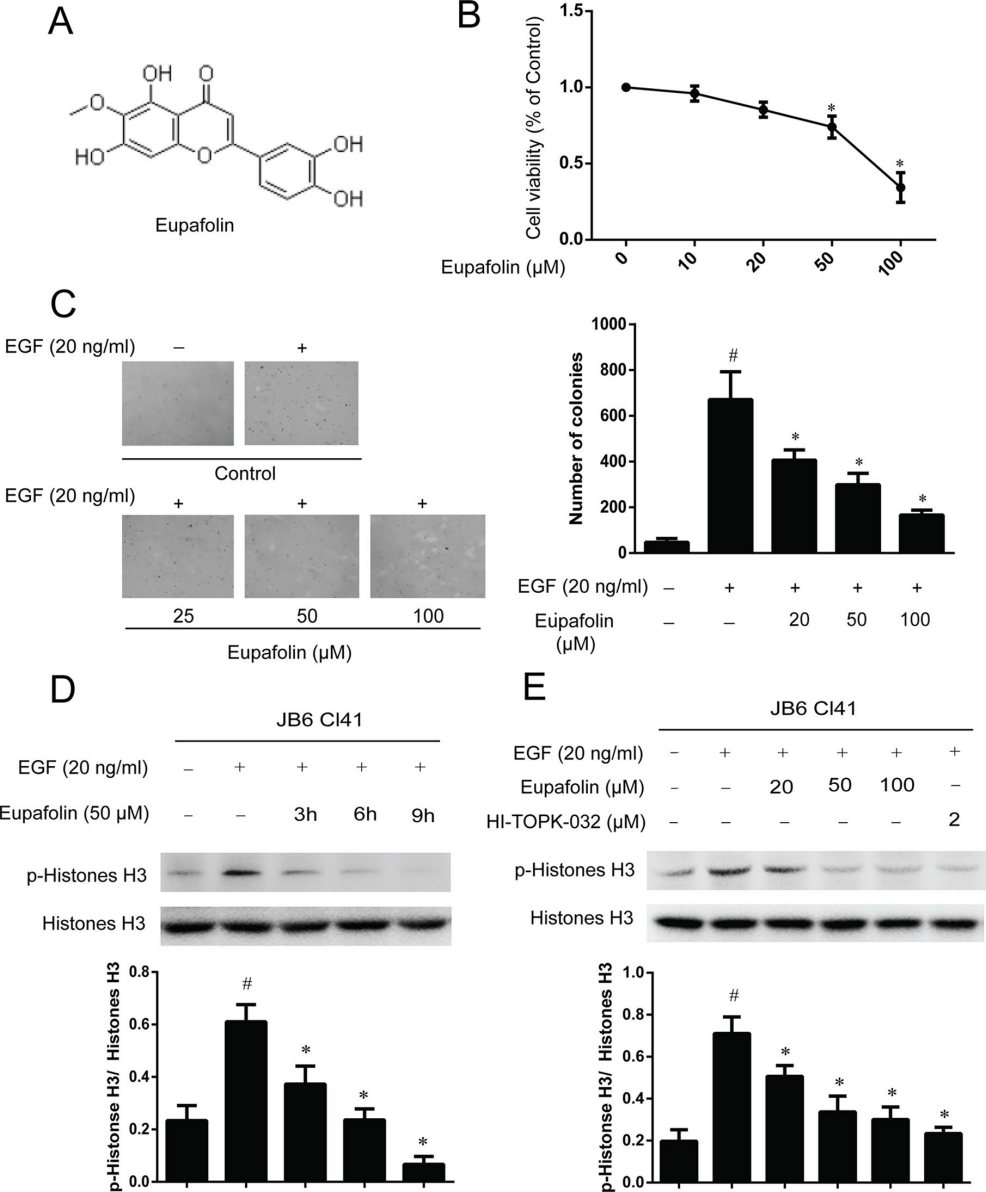
Eupafolin inhibits EGF-induced neoplastic transformation and signal transduction in JB6 Cl41 cells. **(A)** The chemical structure of eupafolin. **(B)** Cytotoxic effects of eupafolin on JB6 Cl41 cells. An MTS assay was used after treatment of JB6 Cl41 cells with eupafolin for 24 h. **(C)** Eupafolin inhibits EGF-induced anchorage-independent growth of JB6 Cl41 cells. JB6 Cl41 cells (8 × 10^3^) were exposed to EGF (20 ng/ml) and treated with eupafolin (0-100µM) in 1 ml of 0.3% Basal Medium Eagle (BME) agar containing 10% FBS, 2 mM L-glutamine, and 25 µM gentamicin. The cells’ colonies were scored using a microscope Motic AE 20 (China). Data are shown as mean ± standard deviation from triplicate experiments. ^#^Significant compared with control alone, P < 0.05. *Significant compared with EGF alone, P < 0.05. **(D)** Eupafolin inhibits TOPK signaling in JB6 Cl41 cells. The cells were starved in serum-free medium for 24 h, then treated in the presence of 50 µM eupafolin for 3, 6, and 9 h, and then treated with 20 ng/ml EGF for 30 min; histones were extracted from cells; total histone H3 and phosphorylated histone H3 proteins were detected by western blot using specific antibodies. Data are representatives of results from triplicate experiments. **(E)** JB6 Cl41 cells were starved in serum-free medium for 24 h, then treated with 20, 50, and 100 µM eupafolin or 2 µM HI-TOPK-032 for 6 h, and then treated with 20 ng/ml EGF for 30 min. The cells were harvested, and protein levels were determined by western blot analysis. ^#^Significant compared with control alone, P < 0.05. *Significant compared with EGF alone, P < 0.05.

The molecular structure of eupafolin was shown in **Figure 2A**. In the present study, we first examined the cytotoxicity of eupafolin in JB6 Cl41 cells by MTS assay. The results indicated that eupafolin did not decrease the viability of JB6 Cl41 cells up to 100 µM at 24 h (**Figure 2B**). Furthermore, we detected the effect of eupafolin on EGF-induced neoplastic transformation of JB6 Cl41 cells. Anchorage-independent growth ability is an *ex vivo* indicator and a key characteristic of the transformed cell phenotype (Freedman and Shin, 1974). Treatment of JB6 Cl41 cells with eupafolin significantly inhibited EGF induced neoplastic transformation in a dose-dependent manner (**Figure 2C**). Eupafolin at 20, 50, or 100 µM decreased 42, 57, or 81% compared to the control group, respectively. These results suggested that eupafolin can reduce the malignant potential of JB6 Cl41 cells induced by EGF.

In the above study, we have found that TOPK is a potential target of eupafolin, and we further detected the downstream signal pathway of TOPK in JB6C141 cells. Western blot results showed that eupafolin suppressed the phosphorylation of histone H3 in a dose- and time-dependent manner, and 2 µM TOPK inhibitor HI-TOPK-032 has the similar effect to 50 or 100 µM eupafolin (**Figures 2D, E**).

### Eupafolin Inhibits Anchorage-Independent Growth of Esophagus Cancer Cells

**Figure 3:**
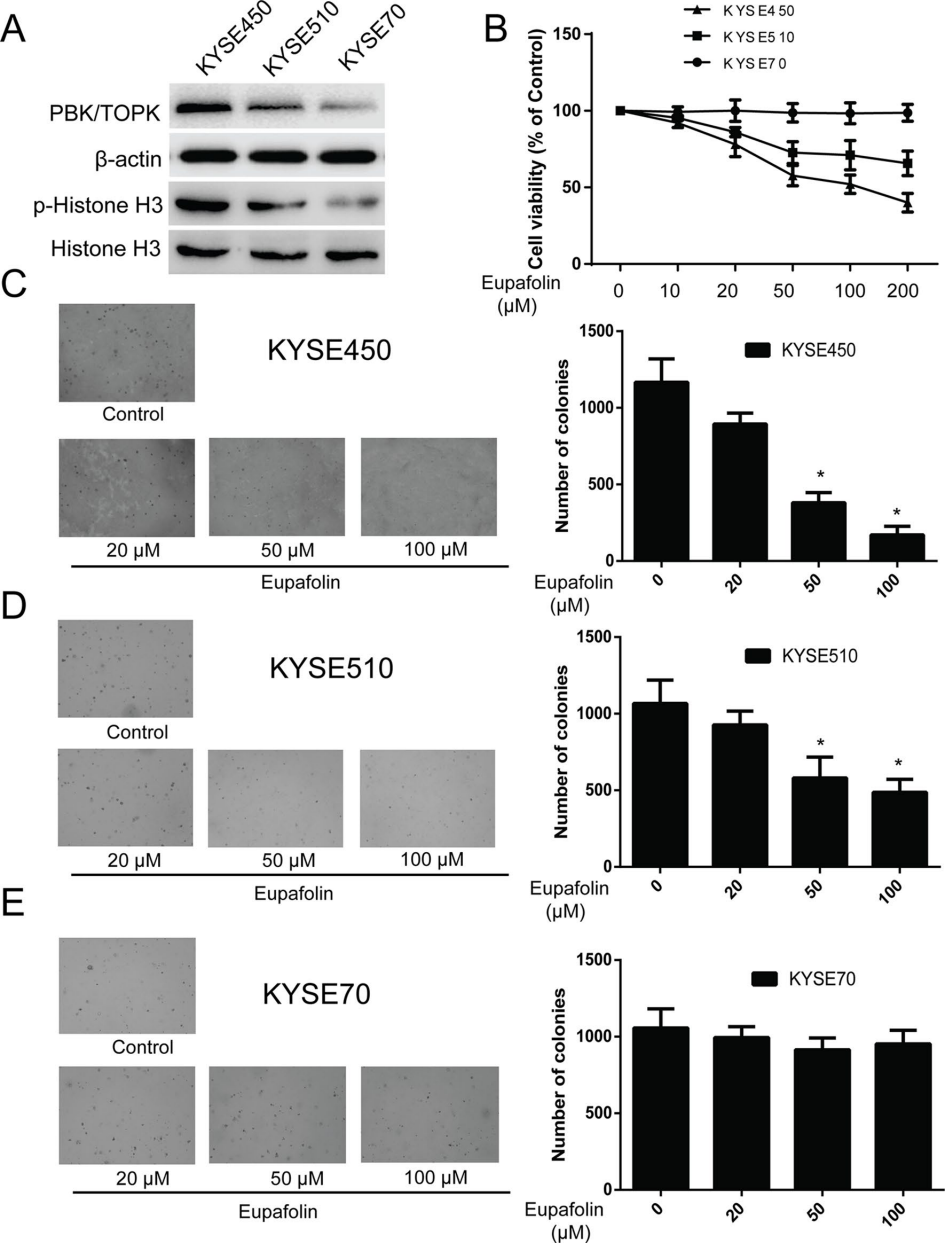
Eupafolin inhibits anchorage-independent growth of esophagus cancer cells. **(A)** Expression of TOPK and p-histone H3 in esophagus cancer cell lines KYSE450, KYSE510, and KYSE70. **(B)** Different concentrations of eupafolin were used to treat the three kinds of esophagus cancer cell lines for 48 h, respectively. Cytotoxicity was measured by MTS assay. **(C**–**E)** The effect of eupafolin on anchorage-independent growth of esophagus cancer cell lines with different level of TOPK expression, including KYSE450 cells **(C)**, KYSE510 **(D)**, and KYSE70 **(E)**. The cells were treated with 20, 50, and 100 µM eupafolin for 2 weeks; then, the number of colonies was scored using a microscope Motic AE 20 (China). Data are shown as means ± standard deviation of values from three independent experiments. *Significant compared with control group, P < 0.05.

Previous studies revealed that TOPK is highly expressed in human esophagus cancer (Ohashi et al., 2016). We attempted to determine whether eupafolin could affect anchorage-independent growth of esophagus cancer cells. We detected the TOPK expression in several esophagus cancer cell lines. We found that TOPK expression was high, medium, and low in three kinds of esophageal carcinoma cells KYSE450, KYSE510, and KYSE70, respectively. At the same time, the trend of p-histone H3 expression was consistent with that of TOPK (**Figure 3A**). Besides, we determine the cytotoxicity of eupafolin by MTS assay. Different concentrations of the drug were used to treat esophagus cancer cell lines KYSE450, KYSE510, and KYSE70 for 48 h, respectively. The results indicated that eupafolin had different cytotoxicity toward different esophagus cancer cells. KYSE450 cells with high TOPK expression were more sensitive to eupafolin (**Figure 3B**). What’s more, neoplastic transformation results showed that eupafolin at 20, 50, and 100 µM inhibited colony formation of KYSE450 cells on 21, 63, and 82%; KYSE510 cells on 12, 35, and 52%; and KYSE70 on 8, 12, and 10% compared with the non-treated cells, respectively (**Figures 3C–E**). Overall, our results suggested that inhibitory effect of eupafolin on colony formation was significant in KYSE450 cells with a high expression level of TOPK.

### Knocking Down TOPK in KYSE450 Cells Decreased the Sensitivity of Eupafolin

**Figure 4:**
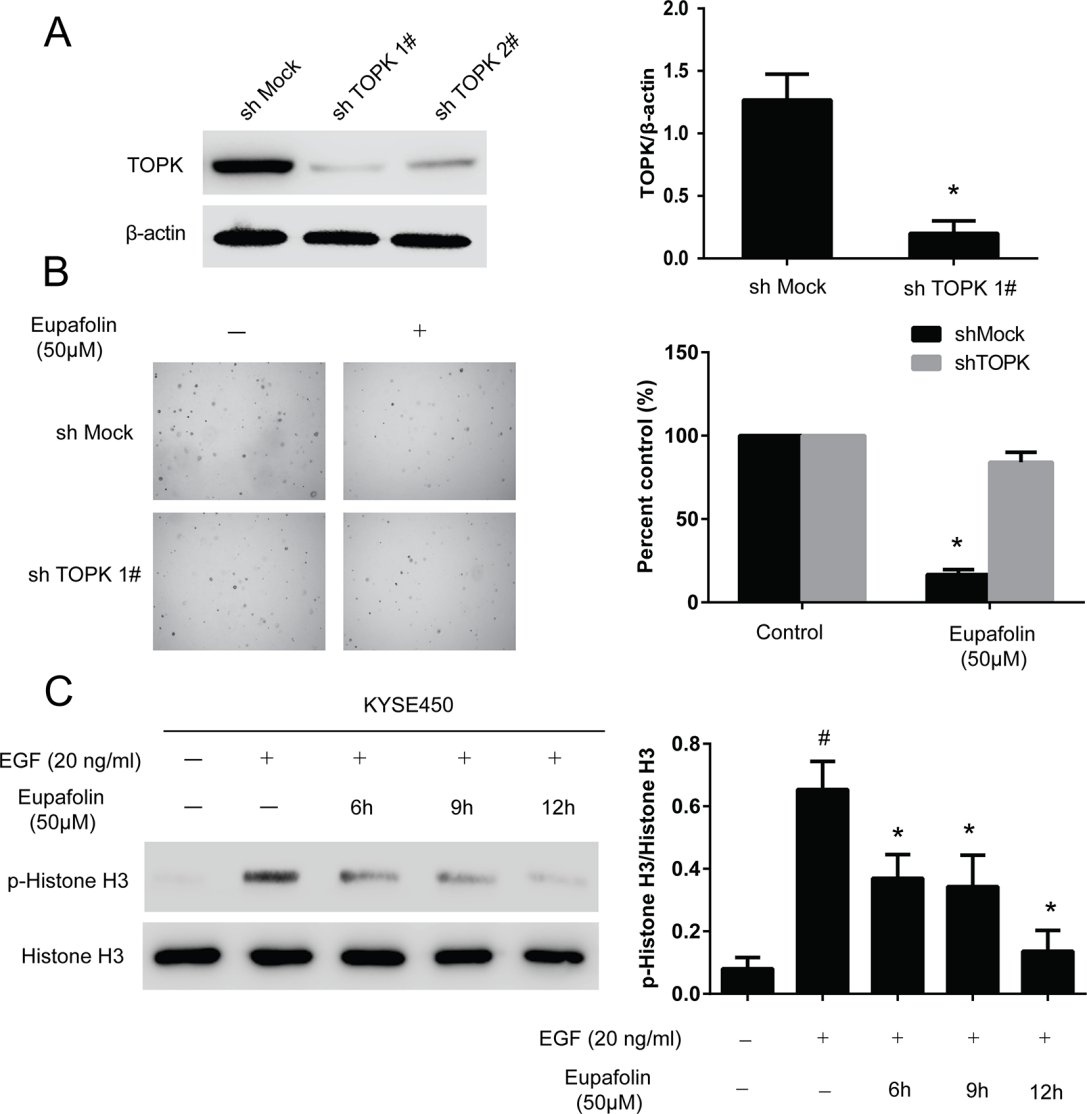
Knocking down TOPK attenuates the inhibitory effect of esophagus cancer cell growth by eupafolin. **(A)** Efficiency of TOPK shRNA in KYSE450 cells. **(B)** Anchorage-independent growth of KYSE450 cells transfected with shMOCK or shTOPK 1#. Data are represented as mean ± standard deviation from triplicate experiments. *Significant compared with control group, P < 0.05. **(C)** Eupafolin inhibits TOPK activity in KYSE450 cells. KYSE450 cells were starved in serum-free medium overnight. Then, the cells were treated with eupafolin (50 µM) for different time then treated with EGF (20 ng/ml) for 15 min. The cells were then harvested, and the protein levels were texted by western blot. Data are representatives of results from triplicate experiments. ^#^Significant compared with control alone, P < 0.05. *Significant compared with EGF alone, P < 0.05.

We then examined whether knocking down TOPK expression influences the sensitivity of KYSE450 cancer cells to eupafolin. Firstly, we determined the efficiency of TOPK shRNA. The results showed that the expression of TOPK obviously decreased after shRNA transfection; the efficient of shTOPK1# was better than shTOPK2# (**Figure 4A**). Then, the growth of cells on anchorage-independent growth assay also decreased over 30% after transfection shTOPK1# compared with the mock group (**Figure 4B**). Moreover, KYSE450 cells transfected with TOPK shRNA1# or mock control were treated with eupafolin or vehicle and subjected to anchorage-independent growth assay. The results showed that eupafolin (20 µM) inhibited colon number of KYSE450 cells transfected with mock shRNA by about 65%. In contrast, the inhibition was only about 17% in KYSE450 cells transfected with shTOPK1#, indicating that KYSE450 cells transfected with shTOPK1# were more resistant to eupafolin treatment (**Figure 4B**). These results suggested that TOPK plays an important role in the sensitivity of KYSE450 cells to the antiproliferative effects of eupafolin. We then investigated the effect of eupafolin on downstream targets of TOPK, the phosphorylation of histone H3 in KYSE450 cells which was relatively more sensitive to eupafolin. Western blot results showed that the phosphorylation level of histone H3 (Ser10) was significantly decreased with eupafolin treatment in a time dependent manner (**Figure 4C**). The above results showed that TOPK is a direct target for eupafolin to suppress esophagus cancer cells growth.

### Eupafolin Suppresses Esophageal Tumor Growth in a PDX Mouse Model

**Figure 5:**
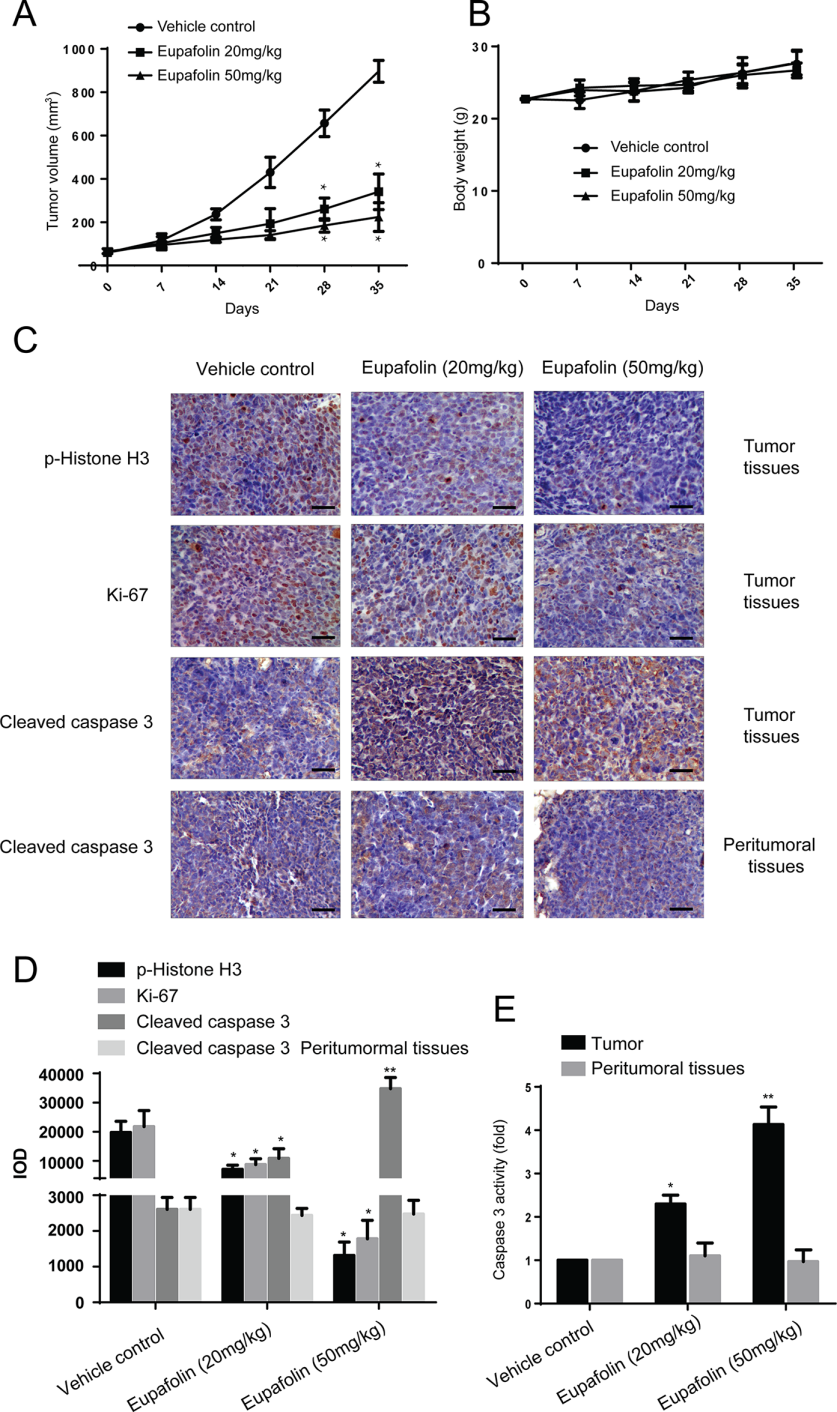
Effect of eupafolin on esophagus cancer growth in PDX mouse model. **(A)** Eupafolin significantly suppresses cancer growth in PDX mouse model. A tumor growth of untreated PDX or treated with different concentration of eupafolin for 35 days. Tumor size was measured once a week and calculated based on the formula: tumor volume = length×width×height×0.5. *Significant compared with control group, P < 0.05. **(B)** Eupafolin has no effect on mouse body weight. Body weights from the treated or untreated groups of mice were measured once a week. **(C)** Eupafolin inhibits expression of phosphorylated histone H3 and Ki67 and increases the expression of cleaved caspase-3 in tumor tissues, and there was no effect on cleaved caspase3 in peritumoral tissues of PDX mouse model. **(D)** Immunohistochemistry analysis was used to determine the expression level of phosphorylated histone H3, Ki67, and cleaved caspase-3 in tumor tissues. **(E)** Eupafolin inhibits the activity of caspase-3 in tumor tissues but had no effect in peritumoral tissues. *Significant compared with control group, P < 0.05. **Signifcant compared with control group, P < 0.01.

Furthermore, to explore the anti-tumor effectiveness of eupafolin in patient-derived xenograft (PDX) with tumor tissues collected from esophageal cancer patients with high expression of TOPK to further investigate the effectiveness of eupafolin. The results showed that eupafolin (20 or 50 mg/kg) effectively inhibited PDX tumor growth compared with the vehicle-treated group (**Figure 5A**) with no significant loss in body weight (**Figure 5B**), suggesting minimal toxicity. Additionally, treatment with eupafolin suppressed phosphorylation of histone H3 expression downregulated the expression of Ki-67 and increased cleaved caspase 3 levels in tumor tissues. While, there were no significant different of cleaved caspase 3 expression in peritumoral tissues (**Figure 5C**). The positive expression of **Figure 5C** was statistically shown in **Figure 5D**. Furthermore, we detected the activity of caspase 3 using Caspase 3 Activity Assay Kit in tumor and peritumoral tissues. The results showed that eupafolin inhibited the activity of caspase 3 significantly in a dose dependent manner in tumor tissues, but it had no effect on the peritumoral tissues (**Figure 5E**). Overall, these results illustrated that eupafolin has potential as chemotherapeutic agent against esophageal cancer.

## Discussion

Esophageal cancer is a kind of malignancies, which is increasing gradually year by year with a higher incidence and mortality. The 5-year survival rate of esophageal cancer is only 10% (Song et al., 2014). Esophageal squamous cell carcinoma and esophageal adenocarcinoma are two kinds of esophageal cancer. Esophageal squamous cell carcinoma accounts for 90% of all cases of esophageal cancer (Smyth et al., 2017) and approximately 70% of esophageal cancer cases occur in China, especially in Anyang city of Henan province (Lin et al., 2017; He et al., 2019).

Chemotherapy is a common method for treating esophageal cancer including 5-fluorouracil (FU), cisplatin, paclitaxel, and mitomycin which are commonly used to treat esophageal cancer as a single treatment or in combination (Chen et al., 2018; Konishi et al., 2018). However, these agents are prone to hematological toxicity (Lyons and Ku, 2017).

Over the past few decades, most of targeted therapies against esophageal cancer has not been progressing as smoothly as hoped, and new targets and inhibitors need to be identified for the treatment of esophageal cancer. TOPK (also known as protein PBK) is a serine-/threonine-specific protein kinase and has been known to audience since 2000 (Abe et al., 2000). Whereafter, a series of discoveries of TOPK function were reported in many kinds of cancers and is involved in various biological processes, including cell proliferation, apoptosis, transcription, migration, and invasion (Hu et al., 2019). It’s reported that TOPK is highly expressed in esophageal cancer and plays an important role in esophageal cancer metastasis (Ohashi et al., 2016). Therefore, TOPK inhibitor could be a promising therapeutic agent for application in esophageal cancer.

Due to the natural compound have higher efficacy and lower toxicity, natural anticancer products have been studied for many years. Eupafolin also is called methoxyluteolin, a flavonoid compound from the Ay Tsao (*Artemisia*). It has shown anti-inflammation (Chen et al., 2016; Zhang et al., 2017), anti-viral (Wang et al., 2013), anti-autism (Patel et al., 2016), anti-angiogenic, and anti-tumor (Liu et al., 2015; Jiang et al., 2017) bioactivities. Eupafolin could be a superior drug for cancer treatment. In our study, we found that eupafolin effectively suppressed anchorage-independent cell growth of esophageal cancer cells with highly expressed TOPK and inhibited growth of patient derived xenograft tumor by suppressing TOPK activities *in vivo*.

In conclusion, eupafolin is a promising therapeutic agent in esophageal cancer chemotherapy by directly targeting TOPK.

## Data Availability Statement

The datasets generated for this study are available on request to the corresponding author.

## Ethics Statement

The animal study was reviewed and approved by Henan Joint International Research Laboratory of Veterinary Biologics Research and Application, Anyang Institute of Technology.

## Author Contributions

XF designed research, performed research and wrote the paper; JT analyzed the data; MF extracted Eupafolin from Ay Tsao; JW expressed Histone H3 protein; XC, ZJ performed animal research and analyzed data; SL, KZ designed research and analyzed data.

## Conflict of Interest

The authors declare that the research was conducted in the absence of any commercial or financial relationships that could be construed as a potential conflict of interest.
